# ThioFinder: A Web-Based Tool for the Identification of Thiopeptide Gene Clusters in DNA Sequences

**DOI:** 10.1371/journal.pone.0045878

**Published:** 2012-09-24

**Authors:** Jing Li, Xudong Qu, Xinyi He, Lian Duan, Guojun Wu, Dexi Bi, Zixin Deng, Wen Liu, Hong-Yu Ou

**Affiliations:** 1 State Key Laboratory of Microbial Metabolism, Shanghai Jiaotong University, Shanghai, China; 2 State Key Laboratory of Bioorganic and Natural Products Chemistry, Shanghai Institute of Organic Chemistry, Chinese Academy of Sciences, Shanghai, China; Wayne State University, United States of America

## Abstract

Thiopeptides are a growing class of sulfur-rich, highly modified heterocyclic peptides that are mainly active against Gram-positive bacteria including various drug-resistant pathogens. Recent studies also reveal that many thiopeptides inhibit the proliferation of human cancer cells, further expanding their application potentials for clinical use. Thiopeptide biosynthesis shares a common paradigm, featuring a ribosomally synthesized precursor peptide and conserved posttranslational modifications, to afford a characteristic core system, but differs in tailoring to furnish individual members. Identification of new thiopeptide gene clusters, by taking advantage of increasing information of DNA sequences from bacteria, may facilitate new thiopeptide discovery and enrichment of the unique biosynthetic elements to produce novel drug leads by applying the principle of combinatorial biosynthesis. In this study, we have developed a web-based tool ThioFinder to rapidly identify thiopeptide biosynthetic gene cluster from DNA sequence using a profile Hidden Markov Model approach. Fifty-four new putative thiopeptide biosynthetic gene clusters were found in the sequenced bacterial genomes of previously unknown producing microorganisms. ThioFinder is fully supported by an open-access database ThioBase, which contains the sufficient information of the 99 known thiopeptides regarding the chemical structure, biological activity, producing organism, and biosynthetic gene (cluster) along with the associated genome if available. The ThioFinder website offers researchers a unique resource and great flexibility for sequence analysis of thiopeptide biosynthetic gene clusters. ThioFinder is freely available at http://db-mml.sjtu.edu.cn/ThioFinder/.

## Introduction

Thiopeptides are a growing family of sulfur-rich and highly modified heterocyclic peptide antibiotics produced in various bacterial strains [1]. This family now contains nearly 100 members, possessing a characteristic macrocyclic core that consists of six-membered monoaza ring central to multiple azoles and dehydroamino acids but varies in side chains (and/or rings) that append additional functionalities. Many thiopeptides inhibit protein synthesis via the mechanisms distinct from clinically used antibiotics. They show potent activity against various drug-resistant pathogens including the methicillin-resistant *Staphylococcus aureus*, penicillin-resistant *Streptococcus pneumonia* and vancomycin-resistant *Enterococcus* species [2]–[3]. Although the poor pharmacokinetics and low water solubility have limited the thiopeptide usefulness in human therapy so far, the interest in this class of antibiotics is recently renewed due to their promising antineoplastic activity in human cancer cells [4]–[5] (Patent: WO2002066046). This further promotes novel analogue generation of thiopeptides for drug development by chemical modification. The complex thiopeptide architecture poses a tremendous challenge to chemical synthesis-based approaches; on the other hand, combinatorial biosynthesis provides a promising way for structural diversity, a prerequisite of which, however, is exploiting the genetic basis of thiopeptide biosynthesis.

Given the remarkable interest in thiopeptide antibiotics and the biosynthetic mechanisms, 11 biosynthetic gene clusters have been reported just in the past four years, including those of thiostrepton (two gene clusters reported) [6]–[7], thiocillins [6]–[8], nosiheptide [9], siomycins [6], nocathiacins [10], cyclothiazomycin [11], thiomuracins [12], GE2270 [12], TP-1161 [13] and GE37468 [14]. The characterization revealed a unifying theme, as the structural complexity of all thiopeptides arises from posttranslational modifications of genetically encoded and ribosomally translated peptides [15]–[16]. Comparative analysis of these biosynthetic gene clusters further underscored the generality that the precursor peptide is subjected to conserved posttranslational modifications to afford the thiopeptide framework [6]–[11], and the specificity that the tailoring proceeds via different routes to furnish individual thiopeptide members [17]–[20].

The post-genomic era now permits to evaluate the potential of thiopeptide production, and gives a slight hint to the metabolite structure with the sequenced biosynthetic gene cluster [21]. Indeed, we have recently demonstrated this applicability by genome mining and consequent confirmation of biosynthetic gene clusters encoding thiocillin in *Bacillus cereus* ATCC 14579 [6] and cyclothiazomycin in *Streptomyces hygroscopicus* 10–22 [11]. We herein report a web-based tool, namely ThioFinder, to rapidly identify thiopeptide biosynthetic gene clusters in the user-supplied nucleotide or genomic sequences. This tool is fully supported by an open-access database, called ThioBase, which documents the substantial information of the known thiopeptides, including the metabolite structure, biological activity, producing organism and target organism, and biosynthetic gene clusters if available.

Bioinformatics resources for targeting identification of bacteriocin biosynthetic gene clusters are available [22]–[25]. Thiopeptide, a newly identified class of bacteriocin, differs greatly from the other known classes by featuring in distinct and more extensive posttranslational modifications during maturation of the precursor peptide [7]. Bacteriocins can be identified and sorted on the basis of a class-exclusive biosynthetic machinery. Current web-based tools such as BAGEL2 [22] are tailor-made for identification of the small genes coding for the other bacteriocins classes, inefficient for thiopeptide biosynthetic gene cluster. Furthermore, there are a few databases including APD2 [23], CAMP [24] and DAMPD [25] featuring collecting and aiding to design antimicrobial peptides; however, they are not specialized to the thiopeptide antibiotics and currently only contain four thiopeptide members (thiostrepton, thiocillin, GE37468, GE2270). In this study, we provide a unique tool ThioFinder for identification of thiopeptide biosynthetic gene clusters from bacterial genome sequences. It employed the multiple features of thiopeptide biosynthetic machinery, including the ribosomal precursor peptide and the highly conserved partners for the thiopeptide-specific posttranslational modifications (cyclodehydration and dehydrogenation, dehydration and hetero-cyclization). Accordingly, the back-end database ThioBase that archived complete information of all 99 known thiopeptide antibiotics and 54 newly identified putative biosynthetic gene clusters was developed. The ThioFinder tool together with ThioBase is believed to facilitate thiopeptide discovery, biosynthetic mechanism characterization, and the application of combinatorial biosynthesis to structural diversification.

## Methods

### ThioFinder, Web-based Tool to Identify Biosynthetic Gene Clusters of Thiopeptides

**Figure 1:**
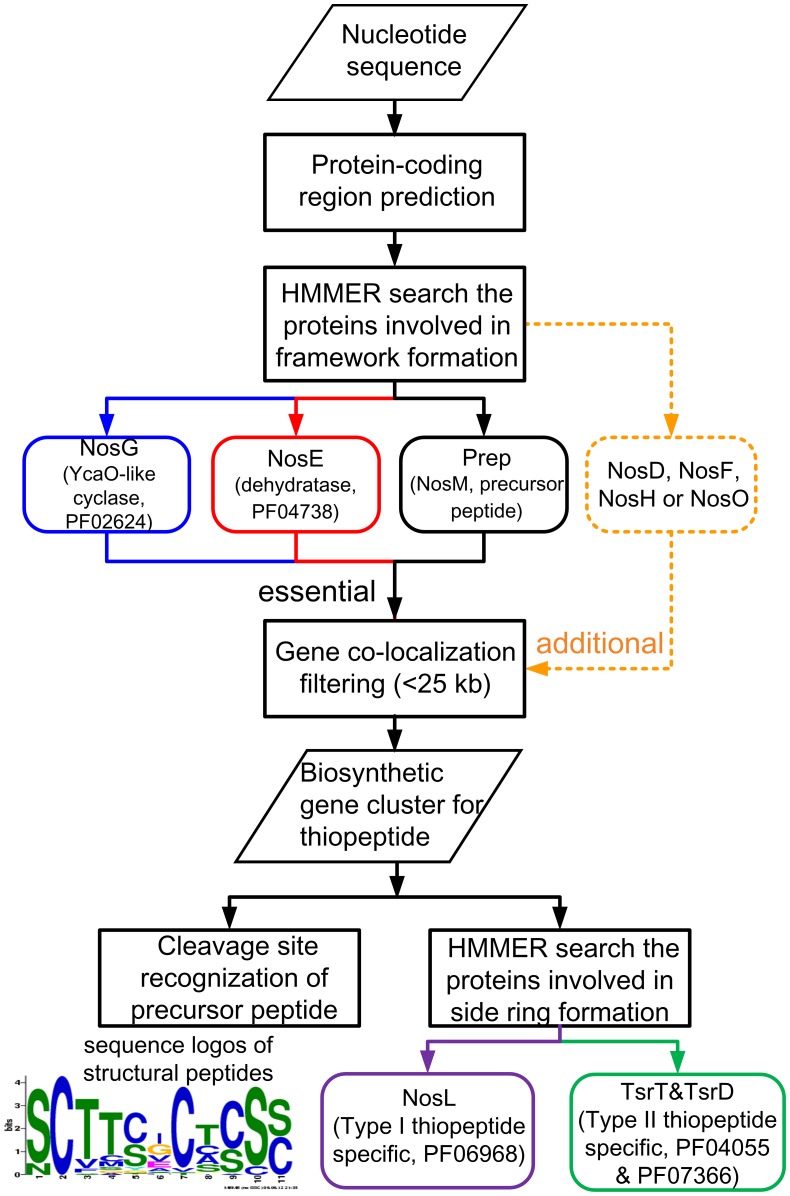
Schematic modular pipeline of ThioFinder.

The online tool ThioFinder utilizes a Hidden Markov Models (HMMs) -based approach to automatically predict thiopeptide biosynthetic gene clusters in the user-supplied nucleotide sequences (Figure 1). In comparison with the frequently used sequence alignment tools based on BLAST-like scoring methodology, the HMMER3 tool employed by ThioFinder can detect the remote protein homologues given the strength of its underlying using probabilistic models (HMM-profiles ) [26]. The highly conserved gene cassette (*thiosf*), involved in thiopeptide-specific framework formation, and the precursor peptide gene (*prep*) were searched in the query nucleotide sequence with the hidden Markov model based profiles (HMM-profiles) summarized in Table 1 **.** Via searches against Pfam 26.0 [27] with the five proteins involved into the nosiheptide biosynthesis as query (NosG, NosE, NosF, NosD and NosL) [9], we easily obtained the HMM-profiles for the five protein families or domains (Table 1): YcaO, Lantibiotic-like dehydratase, Nitroreductase, SpaB C-terminal domain, Biotin and Thiamin synthesis-associated domain. Besides, the HMM-profiles of the NosO-like, NosH-like and Prep-like proteins without significant hit in Pfam were built from 11 known biosynthetic gene clusters of thiopeptides, respectively (marked with the asterisk in Table S1).

**Table 1:** List of the HMM-profiles of the protein families or domains involved in thiopeptide biosynthesis.

Reference protein	Involvement in thiopeptide biosynthesis	Pfam-recorded protein family/domain (accession number)
NosG	azole formation	YcaO, YcaO-like cyclase (PF02624)
NosE	dehydratase	Lant_dehyd_C, C terminus of lantibiotic-type dehydratase (PF04738)
NosF	azole formation	nitroreductase (PF00881)
NosD	dehydratase	SpaB_C, C terminus of SpaB involved in subtilin biosynthesis (PF14028)
NosH	6-membered nitrgen heterocycle	N/A
NosO	6-membered nitrgen heterocycle	N/A
NosL	Type I thiopeptide specific	BATS, biotin and thiamin synthesis associated domain (PF06968)
TsrT	Type II thiopeptide specific	Radical_SAM), radical SAM superfamily (PF04055
TsrD	Type II thiopeptide specific	SnoaL, SnoaL-like polyketide cyclase (PF07366)
Prep	Precursor peptide	N/A

**Figure 2:**
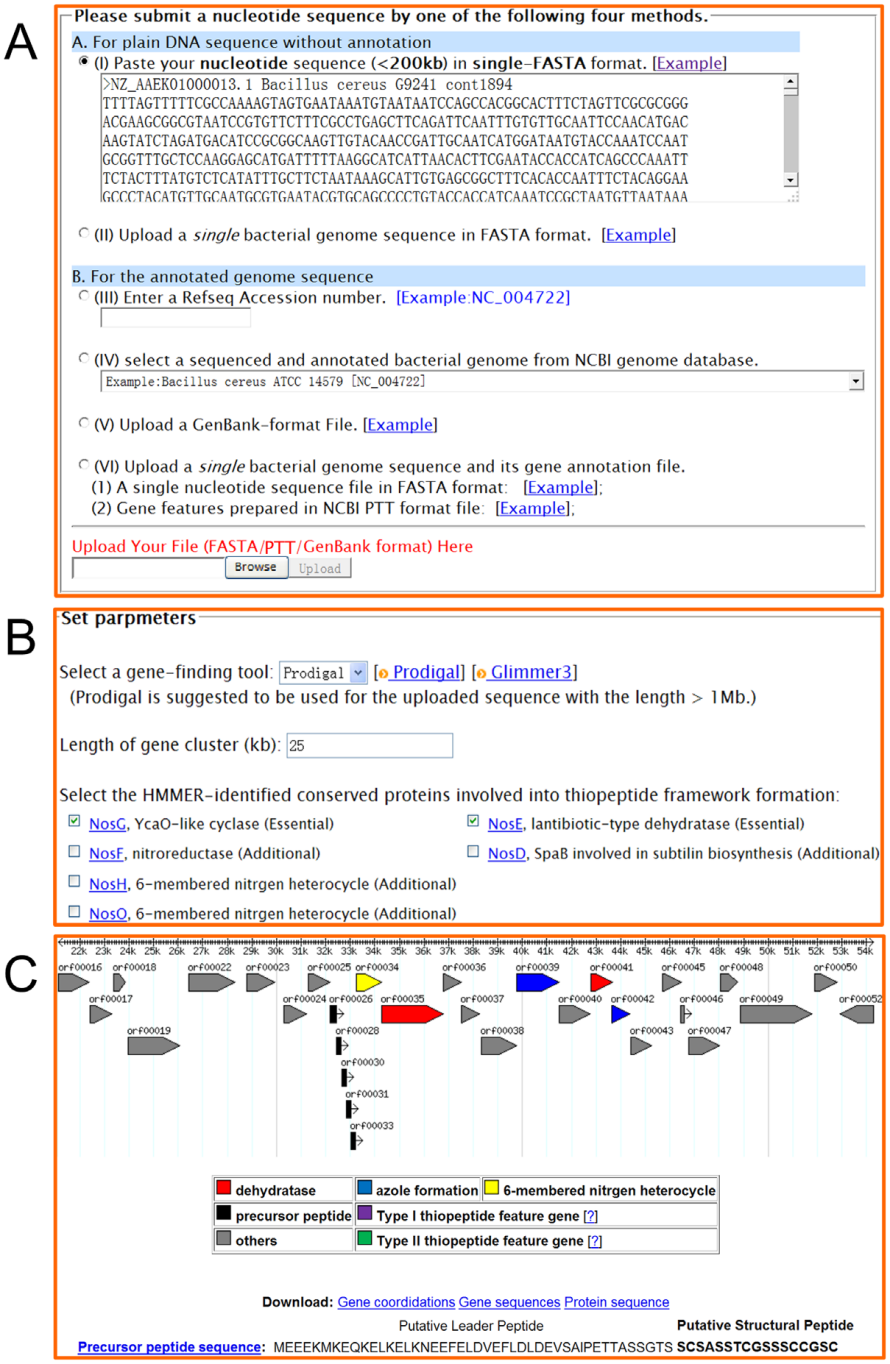
Screenshot and examples of output results of ThioFinder. (A) Uses can upload a raw or annotated nucleotide sequence. Inputs is shown by specifying the contig ‘cont1894’ of *Bacillus cereus* G9241 (NCBI accession no. NZ_AAEK01000013.1). (B) Uses can set various input parameters. Here the protein-encoding regions were identified by using the embedded tool Glimmer3. (**C**) Graphical display for the identified genes coding for the precursor peptide and proteins involved in posttranslational modifications.

In a raw nucleotide sequence (complete genome or scaffold), the ThioFinder tool first identifies the protein-encoding regions with the embedded gene-finding tool Prodigal [28] or Glimmer3 [29] (Figure 2). Users can also upload their own gene annotations. The tool then searches for profiled homologues of thiopeptide biosynthesis by HMMER3::hmmsearch. The region containing co-localized genes encoding the YcaO-like cyclase (homologue of NosG involved in nosiheptide biosynthesis as reference) and the lantibiotic-type dehydratase (homologue of NosE) will be considered as a candidate of thiopeptide biosynthetic gene cluster. The tool then examines the *prep* gene within a flanking 25 kb DNA region. The additional conserved proteins (NosD, NosF, NosH or NosO, if any) coded by the *thiosf* cassette are also searched. Finally, ThioFinder recognizes the putative cleavage sites in precursor peptide sequences. The conserved motif of structural peptide were obtained from 38 known chemical structural thiopeptides by MEME [30]. The sequence logos is shown in Figure 1 and the MEME-defined regular expression for structural peptide is ‘SCTT[CS][GI]CT[CS]S[CS]’. The obtained motif was subsequently used to identify the cleavage site in the precursor peptide sequence by FIMO [31]. A broad range of freely available tools are also integrated into the ThioFinder website to allow for user-directed analyses focusing on thiopeptides or their biosynthetic genes to suit specific interests of the researchers, such as the fast multiple sequence alignment tool MUSCLE [32] and the visualization tool Jalview [33], and the primer design tool Primer3Plus [34].

ThioFinder was developed using Perl and PHP on a Linux platform with an Apache web-server. Web interfaces were developed with HTML, CSS and JavaScript. ThioFinder is now running on a high-performance four-slot four-way server (Inspur NF8560), which is equipped with four six-core XEON E7-4807 1.86 GHz processors and 64 GB Memory.

### ThioBase, Web-based Database to Organize the Known Thiopeptide Information

ThioFinder is fully supported by an open-access database ThioBase. It employs the relational database management system PostgreSQL and runs on the same server of ThioFinder. The majority of data pipelines were developed with PHP and Perl. ThioBase provides a flexible and biologist-friendly web-interface. The homepage mainly contains the following interfaces: ‘Browse’, ‘Search’ (sequence homology search and keyword query by thiopeptides, structural peptides, producer organisms or target organisms), ‘References’ (literatures related to thiopeptides), ‘Introduction’ (description of the thiopeptide characterization and biosynthesis), and ‘Submission’ (report of new thiopeptide or gene cluster to ThioBase). The core of ThioBase is the ‘Browse’ page that provides several organized catalogs (Figure S1), such as ‘Genotype’, ‘gene cluster’, ‘precursor peptide’, ‘nosiheptide genes’ and ‘organism’.

## Results and Discussion

### Typical Features of Biosynthetic Gene Clusters of Thiopeptides

**Figure 3:**
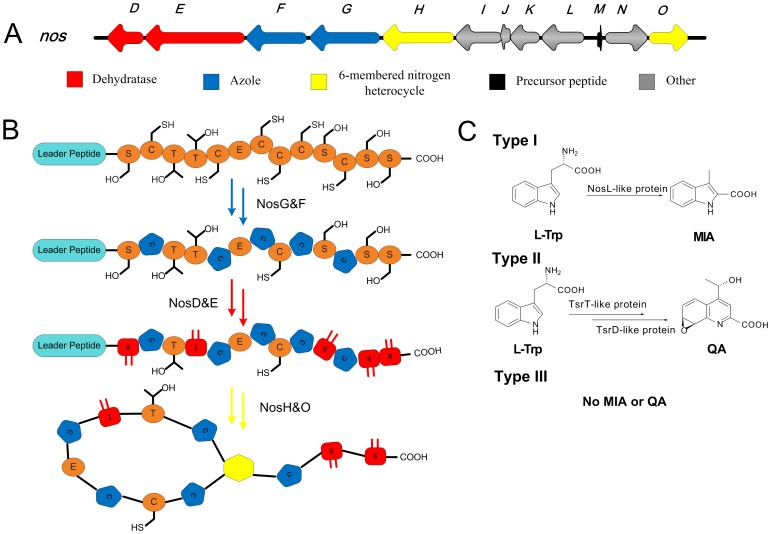
General and specific features of thiopeptidebiosynthetic gene clusters. (A) Organization of the biosynthetic genes, as exemplified by that for nosiheptide utilized as the reference in this study. The red (dehydratases, creating C = C double bonds in seryl, cysteinyl or threonyl residues), blue (azole formation from cysteinyl residues), and yellow arrows (formation of 6-membered nitrogen heterocycle by interaction of two seryl residues) indicate ORFs that have orthologs in all known thiopeptide biosynthetic gene clusters. Small black arrow, gene for the precursor peptide (<100 aa). Some thiopeptide gene clusters contain several (up to5) almost identical precursor peptide genes. Grey, genes that are not conserved between gene clusters, presumably creating the remarkable diversity of thiopeptides. (B) Enzymatic reactions resulting in the highly modified, circular, precursor of a typical thiopeptide. A macro-circular structure, joined at the 6-membered nitrogen heterocycle (yellow) is found in all thiopeptides. Shapes: orange oval with capital letter, natural amino acid; blue pentagon, azole; red square with parallel lines indicating the double bond, dehydroamino acid; yellow hexagon, and six-membered nitrogen ring. Lower case letters indicate the original amino acid residue. (C) Classification of thiopeptide biosynthetic gene clusters. MIA indolyl; QA, quinaldic acid. See Figure 4 for more details.

Thiopeptide biosynthesis typically features one or several almost identical ribosomally synthesized precursor peptides (up to 4 as reported so far) and conserved posttranslational modifications [15]–[16], [35]. Here the *nos* gene cluster of nosiheptide [9] serves as an example (Figure 3). First, the gene cluster contains a *prep* gene encoding a NosM-like precursor peptide less than 120 amino acid residues, whose structural peptide is Cys and Ser/Thr-rich and composes of the resultant thiopeptide backbone. Second, the *prep* gene is co-localized with a highly conserved *thiosf* gene cassette encoding thiopeptide-specific framework formation (Figure 3A). The molecular mechanisms of the framework formation for thiopeptides mainly involve (Figure 3B): (i) a NosG-like cyclodehydratase/NosF-like dehydrogenase complex to produce thiazoles and oxazoles, (ii) a NosD and NosE-like dehydratase pair to form multiple dehydroamino acids. Additionally, a NosO and/or NosH-like homologues may afford the six-membered nitrogen ring. These generality-based criteria for furnishing the thiopeptide-characteristic framework were employed by ThioFinder to identify a gene cluster encodes thiopeptide biosynthesis.

### Identification of Thiopeptide Biosynthetic Gene Clusters from Sequenced Bacterial Genomes

The ThioFinder web server rapidly processes one complete or partial genome sequence per run. Each run is assigned a job ID and the associated output files are stored on the server for 7 days. Presently, ThioFinder takes 2 minutes to identify a thiocillin biosynthetic gene cluster in the 5.4-Mb genome sequence of *Bacillus cereus* ATCC 14579. The detailed reports along with graphical representation are shown in Figure 2.

After running the local version of ThioFinder by command line, we had predicted 65 putative biosynthetic gene clusters of thiopeptides in 1686 complete and 1875 draft bacterial genomes available at NCBI on April 13, 2012 (Table S1). Among them, 11 were known with chemical and genetic experimental results, 54 were firstly reported and pending to be verified whether known or new thiopeptides are produced. Most of the obtained biosynthetic gene clusters of thiopeptides carried both the *thiosf*-like cassettes and the precursor peptide genes *prep,* in agreement with the above criteria for constituting a thiopeptide biosynthetic machinery; whereas 4, in each of which *prep* is missing, only have the *thiosf*-like cassette. The latter suggests that the genetic locus of *prep* can be beyond the 25 kb analyzed DNA region or that the gene clusters are incomplete for thiopeptide biosynthesis. Sometimes, the short gene *prep* (<360 bp) has to be manually checked since it fails to be detected automatically either by the ThioFinder-used gene-finding tool Prodigal or Glimmer3.

To date, 102 organisms belonging to phylogenetically diverse bacterial species are found to harbour thiopeptide biosynthetic gene cluster (see the “Browse by Organism” webpage of ThioBase), including 49 reference-recorded thiopeptide producers and 53 newly identified bacteria based on the searching results of ThioFinder. These strains represented species and genera of variable origin and diverse habitats, such as GC-rich *Streptomyces* versus AT-rich *Bacillus*, natural human vaginal strain *Lactobacillus gasseri* versus human pathogen *Streptococcus pneumoniae,* and *Thermobispora bispora* isolated from decaying manure versus *Verrucosispora maris* from deep marine sediment.

### Information Scope of ThioBase

As of May 25, 2012, the open-access database ThioBase includes the following information. (i) 99 known thiopeptides are listed with the metabolite structures. For each entity, the CAS registry number, analogues, structural peptide sequence, biological activities (antibacterial and/or anticancer data collected from publications and patents), producing organism and hyperlinks to NCBI PubChem are provided. (ii) 65 biosynthetic gene clusters in 63 bacterial species are depicted. 11 of them have been correlated with their coding metabolites, while 54 are newly identified by ThioFinder in the sequenced bacterial genomes currently available at NCBI. (iii) 102 microorganisms are recorded, including 49 reported thiopeptide producers and 53 ThioFinder-predicted bacteria. (iv) Nearly 380 publications relevant to thiopeptides by text mining of NCBI PubMed and SciFinder, which are classified into the following catalogues: ‘isolation and structure characterization’, ‘fermentation and production’, ‘biosynthesis’, ‘biological activity’, as well as ‘chemical synthesis’. Users can query a nucleotide or protein sequence against ThioBase with HMMER3 or BLAST to find homologous matches. The GBrowse viewer [36] was employed for manipulating and displaying annotations on biosynthetic gene clusters of thiopeptides. The ThioBase reference collection is also searchable using the combination of distinct retrieval method, such as by the name of thiopeptide, author, title, journal, year and PubMed ID.

### Relationship between Thiopeptide Biosynthetic Gene Cluster and the Diversified Side Ring System

As the dramatic pace of expansion in bacterial genome data obtained by next-generation sequencing, the putative thiopeptide biosynthetic gene clusters are being identified in increasing numbers by using the *in silico* tools, like ThioFinder. According to the current understanding of thiopeptide biosynthesis, it is becoming feasible to classify the genotypes of thiopeptides, towards establishing the relationship to chemotypes, by taking the specific genes for diversity into account of the resulting structural manners via the biosynthetic reactions.

**Figure 4:**
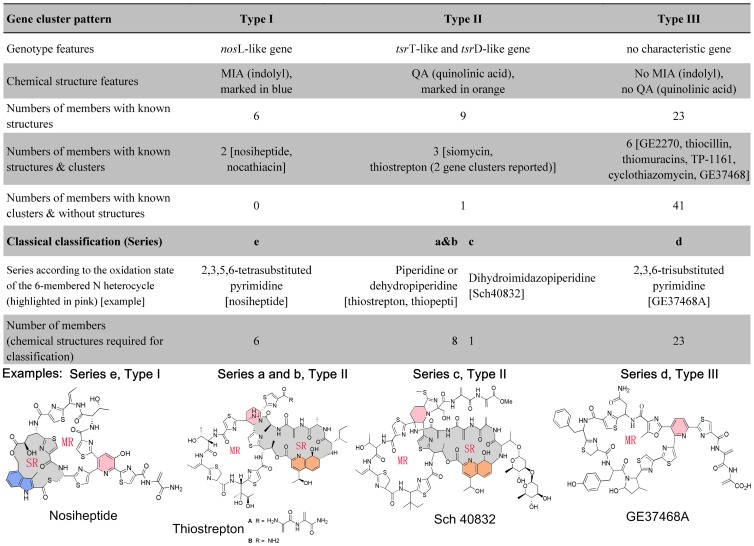
Relationship between biosynthetic gene cluster features and the thiopeptide side ring system. Selected examples of thiopeptides of different types exhibit distinct chemical signatures. MR denotes macrocyclic ring; SR, light grey, side ring. In classical classification**,** Thiopeptides are classified into series a-e according to the oxidative state of the central heterocyclic ring (Pink, 6-membered N heterocycle, including pyrimidine). Three types of thiopeptide gene cluster biosynthetic we proposed makes use of the conserved amino acid sequences of enzyme that produce characteristic indolyl (MIA) or quinolinic acid (QA) residues, which are often found in the side rings of some thiopeptides. Type I, characterized by a side ring containing the indolyl structure (blue, MIA). MIA is synthesized from L-Trp by conserved NosL-like enzymes encoded by one of the grey ORFs. Type II thiopeptides contain side rings with quinaldic acid (orange, QA), which is formed by two enzymes, a hypothetical amidotransferase and a putative ester cyclase for the epoxide ring intermediate. Type III, contains the macro-circular structure but no side ring, and no genes for synthesizing L-trp derivatives (MIA or QA).

We investigated the correlation between the 11 reported biosynthetic gene clusters of thiopeptides and their verified metabolite structures. Three types of thiopeptide biosynthetic gene clusters were characterized (Figure 4): (i) Type I clusters featured a *nosL*-like gene; (ii) Type II characterized a *tsrT*-like and *tsrD*-like genes; (iii) Type III containing non-specific genes. These specific genes are involved in the thiopeptide side ring system in structure, the formation of which is independent of the precursor peptide. Despite sharing a similar macrocyclic framework, the members in thiopeptide family differ in the substitution of the six-membered central ring, installation of the side ring system, decoration of the core system, and C-terminal functionalization of the extended side chain [1] (Figure 4). Biochemical investigations indicated that the functionalization utilizes L-tryptophan as a common substrate but can proceed in completely different ways [37], to afford variable groups as the indolic acid (IA) moiety of nosiheptide and the quinaldic acid (QA) moiety of thiostrepton. As for IA formation, we have recently characterized a radical *S*-adenosylmethionine (SAM) 3-methyl-2-indolic acid synthase (e.g., NosL in nosiheptide biosynthesis) that catalyzes an unprecedented fragmentation-recombination to reconstitute the carbon side chain [20]. By contrast, the QA formation, as that in thiostrepton biosynthesis, involves an unusual methyl transfer (catalyzed by a radical SAM/methylcobalamin-dependent methyltransferase TsrT) onto, and particularly a key ring expansion (involving a cyclase-like protein TsrD) of the indole part [6]–[7], [38]. Comparative analysis of the corresponding gene(s) for L-tryptophan processing among the available 11 biosynthetic gene clusters revealed (i) that formations of IA and QA are common in each moiety-containing bi-macrocyclic members, consistent with the *nosL* homologue for IA in the nocathiacin gene cluster and the *tsrT* and *tsrD* homologues for QA in the siomycin gene cluster, respectively; and (ii) that the gene clusters of the members without the L-tryptophan-derivative moiety, most of which are mono-macrocyclic, apparently lack the above counterparts. These findings supported that the specific gene(s) involved in L-tryptophan processing can serve as a new strategy for classifying the biosynthetic gene clusters of thiopeptides into three types and prediction of the metabolite structures (Figure 4). The correlation of these gene cluster types with their associated chemicals will facilitate new thiopeptide discovery.

The thiopeptides had been classically classified by their chemical structures as five series (a–e), and especially by the oxidation state of the 6-membered nitrogen heterocycle (Figure 4) [1]. Yet this classification cannot be applied to the new thiopeptides predicted from DNA sequence information, long before the substance is characterized chemically. The three gene cluster types we proposed make use of the conserved amino acid sequences of enzyme that produce characteristic indolyl (MIA) or quinolinic acid (QA) residues, which are often found in the side rings of some thiopeptides. Interestingly, when considering the resultant thiopeptide structures, we found the gene cluster types could match to the chemical signatures as well as classical series classification (Figure 4). This suggests the genotypes can be responsible for the chemotypes, and may be deduced from the chemotypes in turn.

### Thiopeptide Side Ring Structure Deduced by the Type-specific Genes

ThioFinder thus grouped the identified gene clusters into the three types along with the products. The 54 newly identified gene clusters were classified into two types,Type II (1 cluster) and Type III (53 clusters). These specific genetic features can hint the structural manners of their potential products. The Type II gene cluster (NCBI accession no. NZ_GG657738), harboring the *tsrT* and *tsrD* counterparts, may encode the biosynthesis of a bi-macrocyclic thiopeptide containing a quinolinic acid moiety. The remaining 53 Type III clusters lacking type-specific gene(s) of L-tryptophan processing may involve in the production of the members without the L-tryptophan-derived side ring.

We also reversely deduced the genetic types of the only structurally known thiopeptides. Of the ThioBase-archived 99 structurally known thiopeptides, 14 were grouped into Type I that features a nosL-like gene encoding IA moiety formation, 21 into Type II that possesses *tsrT* and *tsrD*-like genes for QA moiety biosynthesis, and 64 into Type III that contains none of the above to afford the side ring system. Remarkably, the above genotypes are consistent with the classical thiopeptide classification which is according to the oxidative state of the central heterocyclic domain (Figure 4). The genotypes of Types I and III are completely in line with the thiopeptides of series e (as the monocyclic members with a trissubstituted pyridine central ring) and d (as the bi-macrocyclic members with a hydroxypyridine central ring and an indolic side ring system), respectively. The Type II genotypes are in agreement with the members of series a, b and c, all of which share a piperidine central ring and a QA moiety in the side ring system (Figure 4). The prediction strategy we proposed has the advantage in grouping the genotypes of some members that are structurally almost identical but different only in the central ring, such as thiopeptins, 8 members of which have to be classified into distinct series of chemotypes [1]. Currently the classification is established on the observation of a relative small sample, but it can be further improved by the future characterization of thiopeptide genotypes and chemotypes.

### Conclusions

We have provided a user-friendly interactive tool ThioFinder to quickly and precisely detect thiopeptide biosynthetic gene clusters in the user-supplied nucleotide sequences. The back-end database ThioBase maintains a growing variety of thiopeptide antibiotic related data extracted and curated from experimental literature. Additionally, ThioFinder grouped the identified gene clusters into the three types, towards deducing thiopeptide side ring structure from the type-specific genes. Ultimately, we propose the thiopeptide-specific resource could be of interest to a broad community of the researchers with multidisciplinary backgrounds, to facilitate the further investigation into thiopeptides, the potential candidates in antibacterial and anticancer drug development, both in genetics and chemistry.

## Supporting Information

Figure S1 Organized catalogues on the ‘Browse’ page of ThioBase. (A) List of the known thiopeptides. (B) Detailed information of thiopeptides, regarding the chemical structure, analogue, biological activity, producing strain, biosynthetic gene cluster, structure peptide sequence and reference, as exemplified by that for nosiheptide. Hyperlinks to NCBI PubChem are shown.
(DOC)

Table S1 The list of the thiopeptide gene clusters identified by ThioFinder.
(DOC)
