# Supplementary material for: ThioFinder: A Web-Based Tool for the Identification of Thiopeptide Gene Clusters in DNA Sequences
Source: PLoS One. 2012 Sep 24;7(9):e45878. doi: 10.1371/journal.pone.0045878 (PMC3454323; doi:10.1371/journal.pone.0045878)
Supplement: Figure S1 — Organized catalogues on the ‘Browse’ page of ThioBase. (A) List of the known thiopeptides. (B) Detailed information of thiopeptides, regarding the chemical structure, analogue, biological activity, producing strain, biosynthetic gene cluster, structure peptide sequence and reference, as exemplified by that for nosiheptide. Hyperlinks to NCBI PubChem are shown. (DOC) [file pone.0045878.s001.doc]

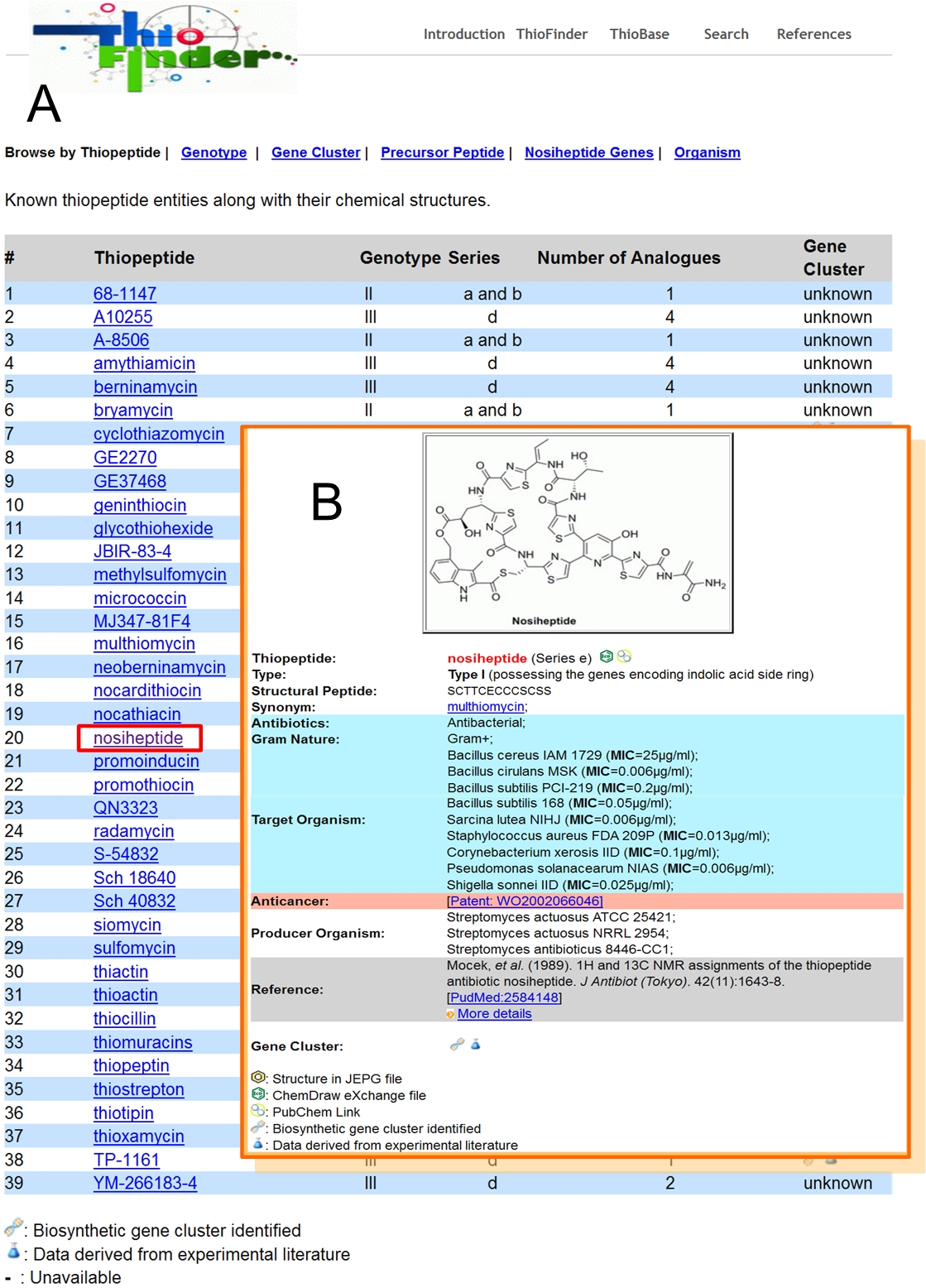


**Figure S1**. **Organized catalogues on the ‘Browse’ page of ThioBase.** (A) List of the known thiopeptides. (B) Detailed information of thiopeptides, regarding the chemical structure, analogue, biological activity, producing strain, biosynthetic gene cluster, structure peptide sequence and reference, as exemplified by that for nosiheptide. Hyperlinks to NCBI PubChem are shown.
