# Supplementary material for: ThioFinder: A Web-Based Tool for the Identification of Thiopeptide Gene Clusters in DNA Sequences
Source: PLoS One. 2012 Sep 24;7(9):e45878. doi: 10.1371/journal.pone.0045878 (PMC3454323; doi:10.1371/journal.pone.0045878)
Supplement: Table S1 — The list of the thiopeptide gene clusters identified by ThioFinder. (DOC) [file pone.0045878.s002.doc]

**Table S1. The list of the thiopeptide gene clusters identified by ThioFinder.**

| # | Organism [NCBI accession no.] | Thiopeptide | Genotype | *Prep no.* | *Thiosf* genes |
| --- | --- | --- | --- | --- | --- |
| 1 | *Actinomadura melliaura* SCC 1655 [DQ297453] ^T^ | - | III | 1 | D E F G H O |
| 2 | *Actinomyces sp. oral taxon* 170 str. F0386 [NZ_AFBL01000091] ^T^ | - | III | 1 | D E F G |
| 3 | *Actinomyces viscosus* C505 [NZ_GL877175] ^T^ | - | III | 1 | D E F G |
| 4 | *Actinosynnema mirum* DSM 43827 [NC_013093] ^T^ | - | III | 1 | D E F G O |
| 5 | *Bacillus atrophaeus* 1942 [NC_014639] ^T^ | - | III | 2 | D E F G |
| 6 | *Bacillus cereus* ATCC 14579 [NC_004722] * | thiocillin I | III | 4 | D E F G H O |
| 7 | *Bacillus cereus* BDRD-Cer4 [NZ_CM000726] ^T^ | - | III | - | D E F G H O |
| 8 | *Bacillus cereus* G9241 [NZ_AAEK01000013] ^T^ | - | III | 5 | D E F G O |
| 9 | *Bacillus cereus* NVH0597-99 [NZ_ABDK02000018] ^T^ | - | III | 5 | D E F G O |
| 10 | *Bacillus sp.* B14905 [NZ_AAXV01000024] ^T^ | - | III | 3 | D E F G O |
| 11 | *Catenulispora acidiphila* DSM 44928 [NC_013131] ^T^ | - | III | 1 | D E F G H |
| 12 | *Catenulispora acidiphila* DSM 44928 [NC_013131] ^T^ | - | III | 1 | D E F G H |
| 13 | *Clostridium cellulovorans* 743B [NC_014393] ^T^ | - | III | 3 | D E F G O |
| 14 | *Corynebacterium diphtheriae* 31A [NC_016799] ^T^ | - | III | 1 | D E F G |
| 15 | *Corynebacterium diphtheriae* BH8 [NC_016800] ^T^ | - | III | 1 | D E F G |
| 16 | *Corynebacterium diphtheriae* C7 [NC_016801] ^T^ | - | III | 1 | D E F G |
| 17 | *Corynebacterium diphtheriae* CDCE 8392 [NC_016785] ^T^ | - | III | 1 | D E F G |
| 18 | *Corynebacterium diphtheriae* HC03 [NC_016787] ^T^ | - | III | 1 | D E F G |
| 19 | *Corynebacterium diphtheriae* INCA 402 [NC_016783] ^T^ | - | III | 1 | D E F G |
| 20 | *Corynebacterium diphtheriae* PW8 [NC_016789] ^T^ | - | III | 1 | D E F G |
| 21 | *Corynebacterium pseudotuberculosis* 316 [NC_016932] ^T^ | - | III | 1 | D E F G |
| 22 | *Corynebacterium pseudotuberculosis* 3/99-5 [NC_016781] ^T^ | - | III | 1 | D E F G |
| 23 | *Corynebacterium pseudotuberculosis* FRC41 [NC_014329] ^T^ | - | III | 1 | D E F G |
| 24 | *Corynebacterium pseudotuberculosis* I19 [CP002251] ^T^ | - | III | 1 | D E F G |
| 25 | *Corynebacterium pseudotuberculosis* P54B96 [NC_017031] ^T^ | - | III | 1 | D E F G |
| 26 | *Corynebacterium ulcerans* 809 [CP002790] ^T^ | - | III | 1 | D E F G |
| 27 | *Corynebacterium ulcerans* BR-AD22 [CP002791] ^T^ | - | III | 1 | D E F G |
| 28 | *Frankia sp.* CcI3 [NC_007777] ^T^ | - | III | 1 | D E F G H O |
| 29 | *Herpetosiphon aurantiacus* ATCC 23779 [NC_009972] ^T^ | - | III | 5 | D E F G H |
| 30 | *Lactobacillus gasseri* JV-V03 [NZ_ACGO02000004] ^T^ | - | III | 1 | D E F G O |
| 31 | *Lysinibacillus fusiformis* ZC1 [ADJR01000053] ^T^ | - | III | 1 | D E F G |
| 32 | *Lysinibacillus sphaericus* C3-41 [NC_010382] ^T^ | - | III | 2 | D E F G O |
| 33 | *Micromonospora sp.* ATCC 39149 [NZ_GG657738] ^T^ | - | II | 1 | D E F G O |
| 34 | *Nocardia sp.* ATCC 202099 [GU564398] * | nocathiacin I | I | 1 | D E F G H O |
| 35 | *Nocardiopsis sp.* TFS65-07 [HM467197] * | TP-1161 | III | 2 | D E F G H O |
| 36 | *Nonomuraea sp.* Bp3714-39 [FJ461360] * | thiomuracins | III | 1 | D E F G H O |
| 37 | *Nonomuraea sp.* WU8817 [FJ461359] * | GE2270A | III | 1 | D E F G H O |
| 38 | *Propionibacterium acnes* HL030PA1 [ADYB01000065] ^T^ | - | III | 1 | D E F G O |
| 39 | *Propionibacterium acnes* KPA171202 [NC_006085] ^T^ | - | III | 1 | D E F G O |
| 40 | *Propionibacterium acnes* SK187 [NZ_ADJM01000020] ^T^ | - | III | 1 | D E F G O |
| 41 | *Propionibacterium sp.* 434-HC2 [NZ_AFIL01000016] ^T^ | - | III | 2 | D E F G O |
| 42 | *Salinispora arenicola* CNS-205 [NC_009953] ^T^ | - | III | 1 | D E F G H |
| 43 | *Salinispora tropica* CNB-440 [NC_009380] ^T^ | - | III | 1 | D E F G H |
| 44 | *Sorangium cellulosum* 'So ce 56' [NC_010162] ^T^ | - | III | - | D E F G |
| 45 | *Streptococcus downei* F0415 [AEKN01000007] ^T^ | - | III | 2 | D E F G |
| 46 | *Streptococcus pneumoniae* INV104 [FQ312030] ^T^ | - | III | 1 | D E F G O |
| 47 | *Streptomyces actuosus* ATCC 25421 [FJ438820] * | nosiheptide | I | 1 | D E F G H O |
| 48 | *Streptomyces albus* J1074 [NZ_DS999645] ^T^ | - | III | 1 | D E F G H |
| 49 | *Streptomyces bingchenggensis* BCW-1 [CP002047] ^T^ | - | III | 1 | D E F G H |
| 50 | *Streptomyces chartreusis* NRRL 12338 [NZ_AGDE01000052] ^T^ | - | III | - | D E F G H O |
| 51 | *Streptomyces griseus* NBRC 13350 [NC_010572] ^T^ | - | III | 1 | D E F G H |
| 52 | *Streptomyces griseus* XylebKG-1 [NZ_GL877172] ^T^ | - | III | 1 | D E F G H |
| 53 | *Streptomyces hygroscopicus* 10-22 [FJ472825] * | cyclothiazomycin | III | 1 | D E F G H O |
| 54 | *Streptomyces laurentii* ATCC 31255 [FJ436358] * | thiostrepton A (A1) | II | 1 | D E F G H O |
| 55 | *Streptomyces laurentii* ATCC 31255 [FJ652572] * | thiostrepton | II | 1 | D E F G H O |
| 56 | *Streptomyces pristinaespiralis* ATCC 25486 [NZ_CM000950] ^T^ | - | III | 1 | D E F G O |
| 57 | *Streptomyces sioyaensis* ATCC 13989 [FJ436355] * | siomycin A | II | 1 | D E F G H O |
| 58 | *Streptomyces sp.* AA4 [NZ_GG657747] ^T^ | - | III | 1 | D E F G |
| 59 | *Streptomyces sp.* ATCC 55365 [JN052143] * | GE37468 | III | 1 | D E F G H O |
| 60 | *Streptomyces sp.* C [NZ_GG657750] ^T^ | - | III | 1 | D E F G H |
| 61 | *Streptomyces sp.* NRRL 30471 [HQ257512] ^T^ | - | III | 1 | D E F G H O |
| 62 | *Streptomyces venezuelae* ATCC 10712 [FR845719] ^T^ | - | III | 2 | D E F G H O |
| 63 | *Thermobispora bispora* DSM 43833 [NC_014165] ^T^ | - | III | 1 | D E F G H O |
| 64 | *Verrucosispora maris* AB-18-032 [NC_015434] ^T^ | - | III | 2 | D E F G H |
| 65 | *Weissella paramesenteroides* ATCC 33313 [NZ_ACKU01000026] ^T^ | - | III | - | E G O |

### * Known biosynthetic gene clusters ^T^ Thiopetide gene clusters identified by ThioFinder - undetectable
